# Type 2 diabetes education programmes – focusing on patients’ perceptions

**DOI:** 10.1186/1757-1146-8-S1-A13

**Published:** 2015-04-20

**Authors:** Julie Hastings, Lisa Chandler

**Affiliations:** 1 The University of Northampton, Northampton, UK

## Objective

The prevalence of Type 2 Diabetes in the UK during 2013 was 0.6%, equating to 3.2 million people. Forecasts indicate that three people will be diagnosed every ten minutes by 2025. Ineffective methods or delays in thorough patient education leaves the patient at risk of developing foot complications among wider issues of morbidity. This research aimed to test the efficacy of patient education for Type 2 Diabetes by analysing patients’ views following their educational programme. The project aimed to highlight the value of such education programmes and to encourage wider holistic awareness of education in patient management.

## Method

With School of Health ethical committee approval, a focus group was formed using a random sample of seven consenting Podiatry patients from a UK School of Podiatry. All patients had a diagnosis of Type 2 Diabetes and had attended an educational programme at some point over the last 12 years. The researcher initiated discussion with questions to the group and a one hour discussion was transcribed. This was defined using Nvivo 10 software and thematic analysis was employed to look at quality, recurrent themes and their significance in relation to the existing evidence base. The primary outcome measure was comprehension. Positive and negative perceptions were defined into subthemes to measure specific outcomes.

## Outcome

This qualitative research study considered the efficacy of educational programmes for patients with a diagnosis of Type 2 Diabetes, using patients’ perceptions. Contexts around stabilisation of glucose levels, quality of life, patient adherence and relationships were analysed. Although diet was quantifiably discussed, reference to comprehension and consistency of patient education were defined in order to answer the research question. Compared to current evidence, measures of group versus individual education, biomedical outcomes in the disease process and perceptions via questionnaires, this study found structure of education programmes to be the underlying key to successful outcomes.

## Discussion

Effectual education is reliant upon collaborative interventions of all Health Care Professionals, so that consistent information is delivered and patients get the practical skills required to self-manage their Diabetes. Furthermore, patients’ knowledge of their condition plays a valuable adjunct to basic awareness of Diabetic foot disease. Though there are many programmes available, it is evident that some patients newly diagnosed and some with long standing Type 2 Diabetes have limited knowledge of their condition. They feel anxious and confused, however; the majority of patients embrace the chance to share their story and knowledge, feeling empowered upon doing this.

This study has served as a useful pilot study and compared existing literature with the presenting qualitative evidence, however; for future research in this area a wider study is recommended to further investigate patient trends in a larger population. Moreover recommended are all practitioners involved ensure patient education forms part of a treatment plan and that we ensure, as professionals, we constantly question our practice. How do we educate best, and are the messages consistent in our delivery of patient education?

